# Unique Presentation of Glioblastoma With Acute Onset Symptomatology and Disease Recurrence

**DOI:** 10.7759/cureus.81872

**Published:** 2025-04-08

**Authors:** Angelo Federico, Alexander A Restum, Chadi Faraj

**Affiliations:** 1 General Surgery, Corewell Health, Dearborn, USA; 2 Medical Education, Wayne State University School of Medicine, Detroit, USA

**Keywords:** cns tumor, glioblastoma multiforme, grade 4 astrocytoma, neurology, perfusion mri, radiation oncology

## Abstract

Glioblastoma multiforme (GBM) is the most common primary brain tumor in adults. This devastating disease is well known for both its poor prognosis and rapid disease progression. Associated risk factors range from increasing age to certain predisposing genetic aberrations. Despite advances in surgical, therapeutic, and immunological therapies, there has been little improvement in the overall survival rate, and GBM remains an incurable disease. Here, we report the unique case of a 54-year-old female patient who was ultimately diagnosed with GBM after presenting with a very rapid onset of symptoms and discuss both the variability in the presentation of glioblastoma and the importance of monitoring for disease recurrence.

## Introduction

Within the United States, malignant primary brain tumors account for approximately 15,000 deaths per year and have a five-year overall survival rate of 67%; glioblastomas account for 49% of these brain tumors [1]. Deemed the most aggressive and malignant type of astrocytoma according to the World Health Organization (WHO) classification, GBM is considered a grade IV malignancy and carries an expected 5-year survival rate of only 7.2% [2]. The incidence rate is 3.19 per 100,000, with the primary diagnosis occurring in individuals with a median age of 64 [3]. Over 90% of diagnosed GBM cases are primary gliomas, arising from normal glial cells through multistep oncogenesis, while the remaining cases are secondary gliomas and originate from tumors of lower grades [4]. These secondary cases characteristically grow more slowly and have an overall better prognosis. The incidence of GBM is higher in Caucasian individuals, specifically Caucasian men living in industrial areas [3,4]. Interestingly, there has been no substantial evidence supporting the association of GBM with specific lifestyle characteristics, such as tobacco use or alcohol consumption [3].

The clinical presentation of the disease may vary widely and is dependent on numerous variables, such as tumor location and size at the time of diagnosis. The most common presenting symptoms at the time of diagnosis are typically headache and nausea, secondary to a large tumor size or a significant amount of edema [5]. Other presentations include sequelae of intracranial hypertension, motor deficits, confusion, and more. While these symptoms typically have a gradual onset and lead to a diagnosis within weeks, this delayed diagnosis can lead to a worsened prognosis, reduced survival time, and a decline in quality of life secondary to the aggressive and rapid growth of the tumor. This report discusses a case with an acute onset of symptomatology with prompt diagnosis and operative intervention.

## Case presentation

The patient is a 54-year-old African American woman with a past medical history of hypertension. Her social history included occasional tobacco use and social alcohol consumption. She initially presented to the emergency department (ED) with concerns of stroke-like symptoms. She stated that earlier in the day, she began to experience an acute onset of right-sided facial droop, as well as dysarthria that she described as slurred speech and excessive drooling. She denied experiencing confusion, gait instability, headache, vision changes, weakness, or other neurological symptoms. She denied any prior episodes of similar symptoms as well. After symptom onset, the patient went immediately to the ED.

**Figure 1:**
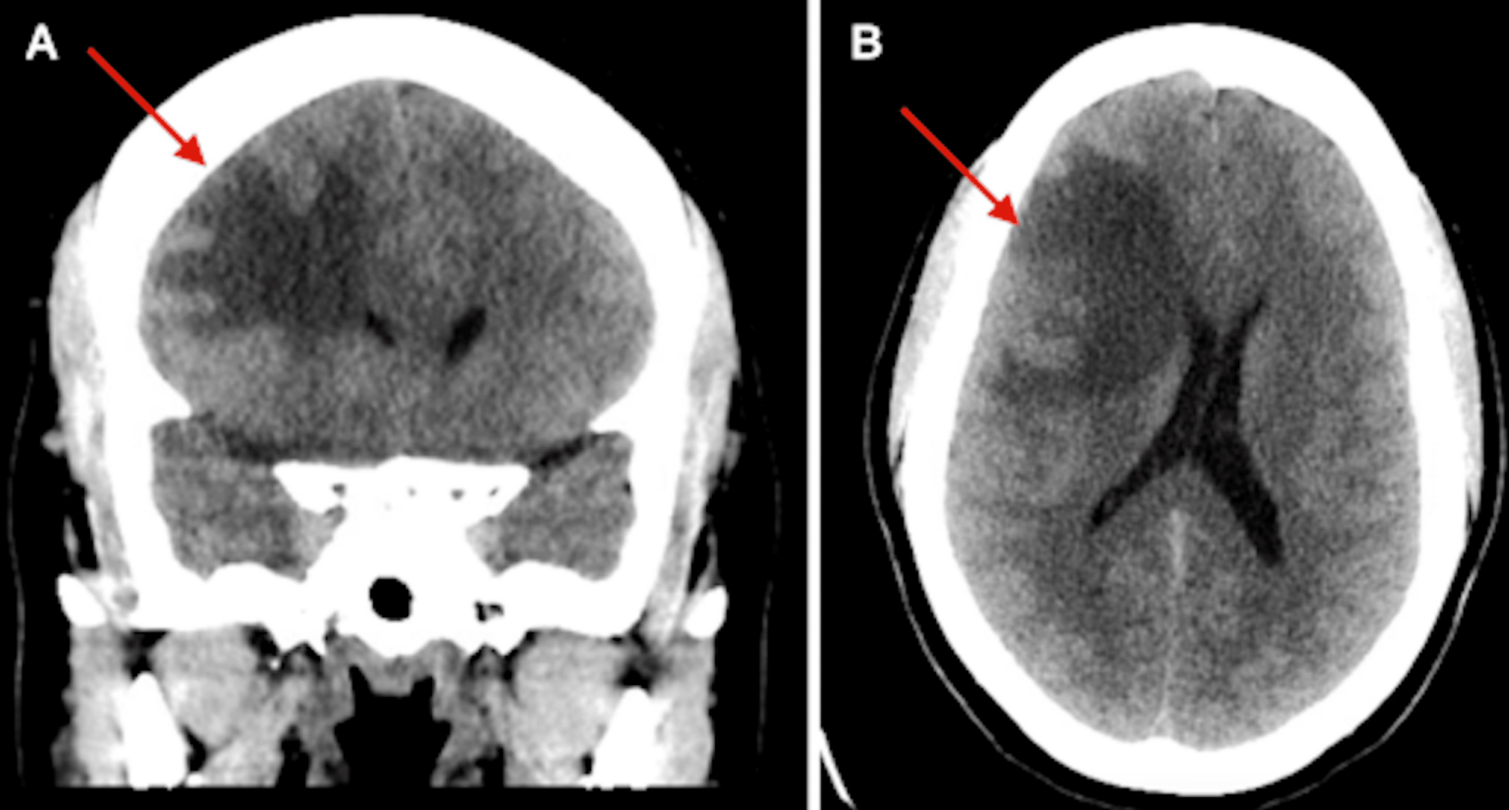
CT head showing a 3.6 cm mass (red arrows) within the right frontal lobe with associated vasogenic edema and mass effect on the right lateral ventricle. (A) Coronal view demonstrating mass effect on right lateral ventricle. (B) Axial view demonstrating 6 mm of leftward midline shift.

Upon arrival, that patient had complete resolution of her symptoms; she was appropriately alert and oriented with no evidence of neurological deficits. Given her prior symptomatology, a computed tomography (CT) scan of the head was performed. This scan demonstrated a 3.6 cm mass within the right frontal lobe with a large amount of associated vasogenic edema, along with mass effect on the right lateral ventricle with 6 mm of leftward midline shift (Figure 1). No evidence of acute hemorrhage or extra-axial fluid collection was noted.

**Figure 2:**
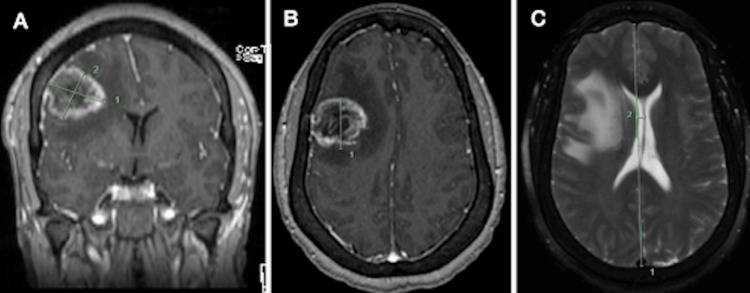
MRI brain demonstrating a 3.5 x 3.0 x 3.3 cm mass centered within the right frontal opercular region with a large amount of surrounding vasogenic edema, leading to approximately 5 mm of leftward midline shift with slight effacement of the right lateral ventricle. (A) Coronal view demonstrating the mass in the right frontal opercular region. (B) Axial view demonstrating the mass with surrounding vasogenic edema. (C) Axial view showing 5 mm of leftward midline shift.

Given the mass visualized within the frontal lobe, the neurosurgery team was consulted. The patient was started on dexamethasone secondary to cerebral vasogenic edema and levetiracetam for seizure prophylaxis. A CT of the thorax, abdomen, and pelvis was performed to evaluate for metastatic disease and was negative for acute pathology. A magnetic resonance imaging (MRI) scan of the brain was ordered for further characterization of the mass and was subsequently completed the day after initial presentation. This study revealed a heterogeneously enhancing 3.5 x 3.0 x 3.3 cm mass within the right frontal opercular region with a large amount of surrounding vasogenic edema causing approximately 5 mm of leftward midline shift with slight effacement of the right lateral ventricle (Figure 2). The involvement of the gray-white junction was also noted. Given these imaging findings, a right-sided craniotomy for resection of the mass was scheduled for the following day.

**Figure 3:**
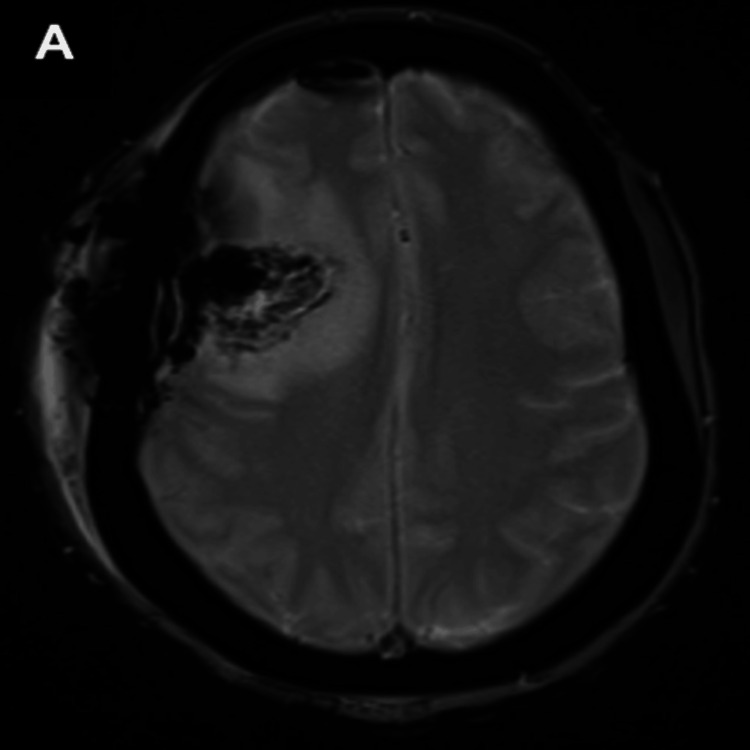
MRI showing interval post-surgical changes with fluid within the resection cavity and a thin rind of cytotoxic edema around the cavity. No overt residual mass identified and leftward midline shift slightly improved.

During the procedure, the tumor was identified in the cranial region near the temporalis muscle and confirmed using stealth navigation. The mass was carefully dissected using bipolar cautery and microscissors, with further dissection aided by blue light and Sonopet (Stryker, Kalamazoo, MI) to indicate positive tumor tissue. All visible tumor tissue was meticulously removed. Following completion of the procedure, the patient was admitted to the intensive care unit for frequent neurological monitoring and appropriate Gleolan precautions. She progressed well postoperatively without any apparent neurologic deficits and was subsequently discharged on postoperative day four. Given the presence of cerebral edema, the patient was discharged on a dexamethasone taper. Postoperative MRI demonstrated interval post-surgical changes with fluid within the resection cavity and a thin rind of cytotoxic edema around the cavity (Figure 3). No overt residual enhancing mass was identified, and the previously visualized leftward midline shift was noted to be slightly improved, from 5 mm to 2 mm. 

The resultant pathology from the resection demonstrated a high-grade glioblastoma, IDH-wild type, CNS WHO grade IV. Molecular features of the glioma included IDH-negative, ATRX retained, p53 positive, and MGMT methylation negative. Given this new diagnosis, the patient was instructed to follow up with oncology to establish care and create a treatment plan. Shortly following discharge, the patient was started on concurrent radiation therapy (RT) and temozolomide with plans for maintenance temozolomide following completion of the concurrent therapy. The patient tolerated RT and temozolomide well and completed the concurrent course. During the interim period before starting maintenance temozolomide, the patient presented to the ED with concerns of seizure-like activity. She was noted to be postictal upon arrival but shortly recovered to her neurologic baseline. 

**Figure 4:**
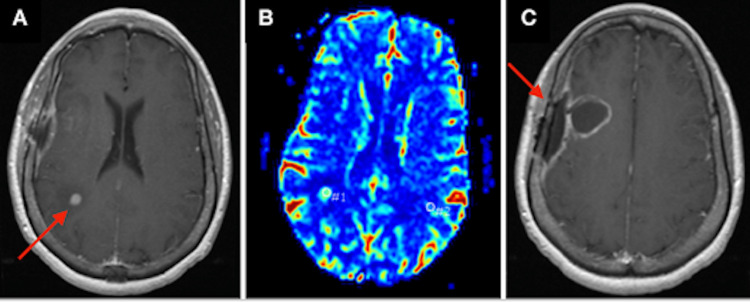
MRI brain showing interval development of an 8 mm rim-enhancing mass in the deep white matter of the right parietal lobe with extensive vasogenic edema. MR perfusion scan shows a significant increase of MR perfusion compared to normal brain, consistent with a new brain metastasis. (A) Axial view of the 8 mm rim-enhancing parietal mass (red arrow). (B) Axial view of MR perfusion scan showing increased perfusion of the mass. (C) Axial view demonstrating postoperative changes with a right frontal parietal craniotomy (red arrow).

Given this new symptomatology, an MRI brain was performed and again demonstrated expected post-surgical changes at the previous resection cavity, but now also demonstrated an 8 mm round, rim-enhancing lesion with surrounding vasogenic edema in the right peritrigonal area (Figure 4).

**Figure 5:**
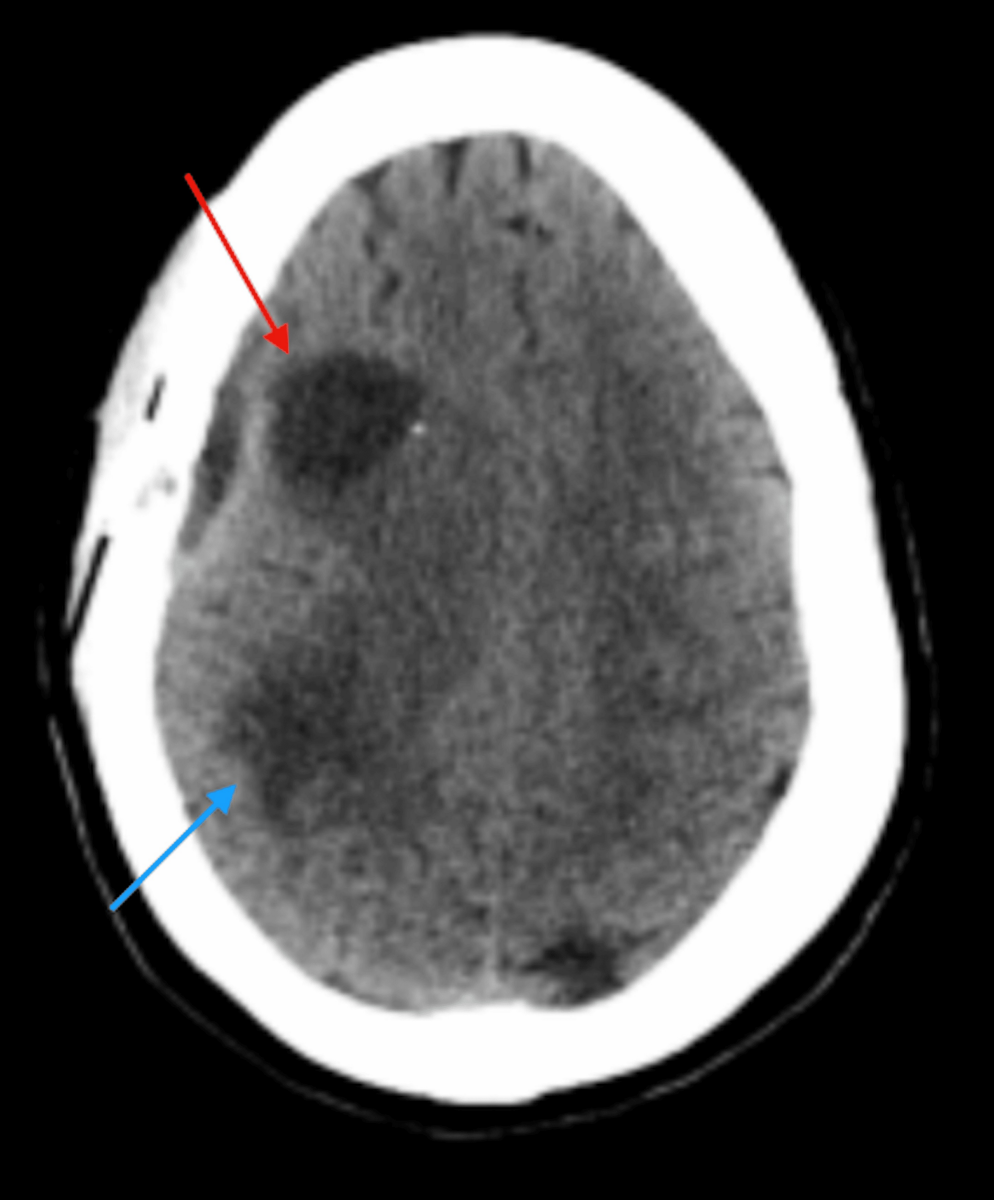
CT of the head revealing a new ill-defined 2.6 cm area of right peritrigonal white matter edema (blue arrow) and post-surgical changes with a 2.3 x 2.6 x 2.4 cm fluid collection in the right frontal resection cavity (red arrow).

Given this finding, there was high clinical suspicion for a new brain metastasis. A CT of the head was performed and revealed interval development of ill-defined 2.6 cm right peritrigonal white matter edema compared to imaging from her index presentation (Figure 5).

**Figure 6:**
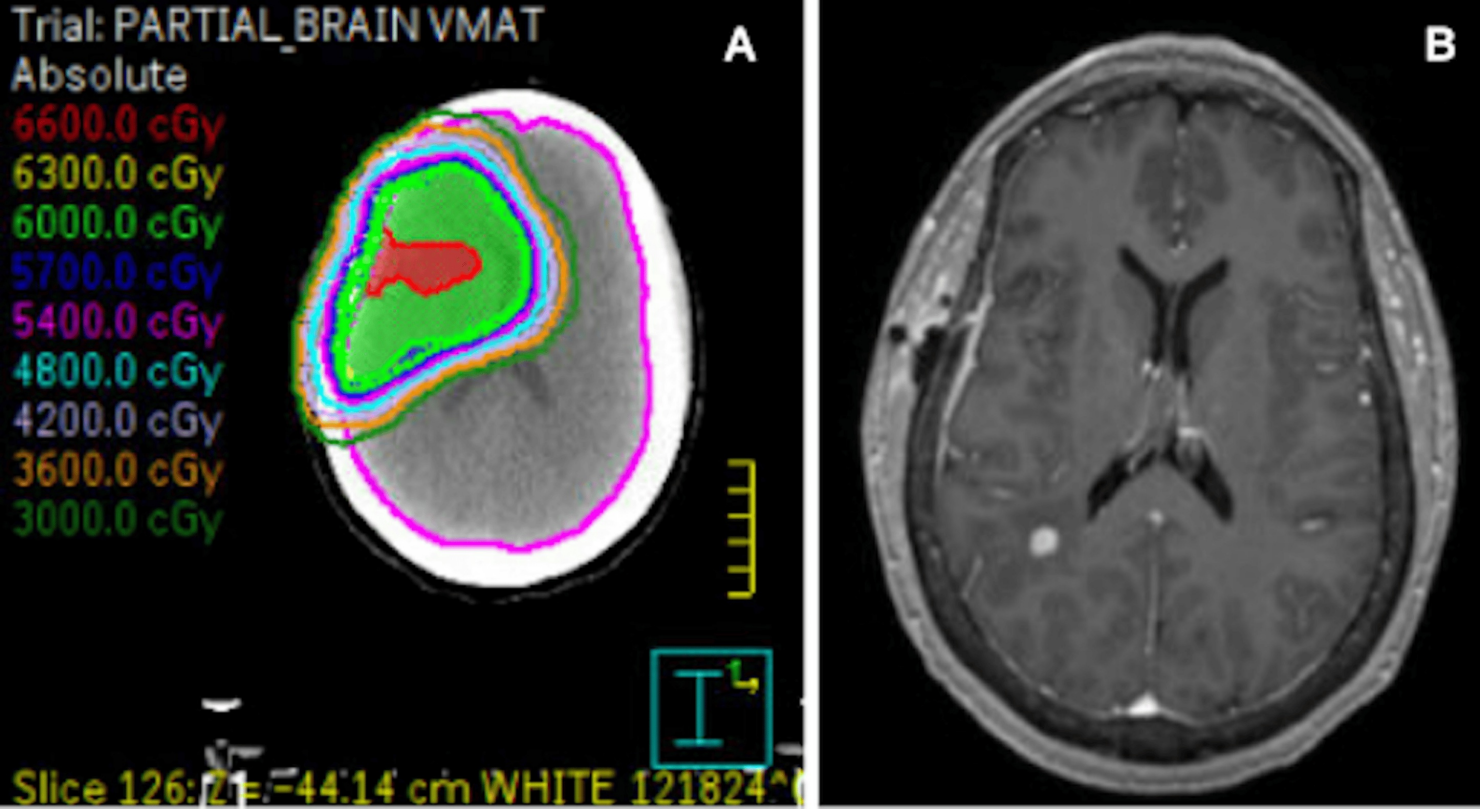
Prior radiation field (A) and tumor location on MRI (B).

Given that the patient was chronically immunosuppressed and recently had undergone radiation, potential clinical concerns included a cerebral abscess, radiation necrosis, and a new metastasis. The case was subsequently discussed at a multidisciplinary tumor board. The clinical suspicion for an abscess was low, given the uncommon anatomical location and lack of an obvious source of infection. The suspicion for radiation necrosis was also felt to be relatively low, given that the lesion site was fairly removed from the tumor bed and was outside the prior radiation field, receiving less than 30 Gy (Figure 6). In terms of treatment options, surgery was not recommended based on the small size of the lesion. Given the focal nature of the recurrence, gamma knife stereotactic radiosurgery (GK-SRS) as a therapeutic option was discussed. Further information regarding the patient’s treatment course and disease progression is unavailable as, following this ED visit, the patient relocated to a different state to be closer to her social support system and, therefore, established care with a new medical oncologist.

## Discussion

GBM is the most common and aggressive primary central nervous system malignancy and has an estimated median survival of only 15 months [3]. Despite the research efforts to identify genetic and environmental factors associated with the disease, most are sporadic and there have not been any major risk factors identified [3]. Despite this lack of risk factor identification, there have been favorable clinical prognostic factors that have been identified. These include younger age at the time of diagnosis, cerebellar tumor location, high patient performance status, and maximal tumor resection [3].

The treatment for newly diagnosed glioblastoma is multifaceted and complex. Initial treatment typically consists of maximal-safe surgical resection of the tumor, followed by RT and concurrent chemotherapy. This course is then followed by further maintenance chemotherapy. The chemotherapy agent of choice for GBM is temozolomide, which is an alkylating agent. The addition of temozolomide is associated with both increased overall survival and progression-free survival, and when used concomitantly with radiotherapy, the median survival time is noted to be two months greater than when radiotherapy is used for treatment alone [6]. 

During follow-up of patients undergoing RT and chemotherapy, an MRI scan is typically performed every two to three months [7]. The completion of regular MRI scans can aid in the detection of disease recurrence in the early phase, often prior to the onset of clinical manifestations. This imaging is sometimes performed sooner if the patient starts to experience clinical deterioration, such as our patient. The interpretation of follow-up MRI scans to make a radiological diagnosis of recurrence can be difficult, however, given the possible pseudoprogression, pseudoresponse, or radionecrosis in those that have undergone RT and chemotherapy [8]. Unlike a newly diagnosed glioblastoma, the standard of care for patients with a recurrence, such as our patient, remains highly debated. Reoperation can be considered to extend the overall survival based on certain tumor factors, such as location, size, patient performance status, and time from initial diagnosis. It is estimated that only about 25% of patients with a recurrence are considered for repeat debulking surgery [8]. The key factor for the outcome and prognosis for those undergoing reoperation is the extent of resection of the relapsed tumor [8]. For our patient, however, operative resection was not recommended based on the small size of the lesion.

## Conclusions

In the case described above, the patient initially presented with acute onset right-sided facial droop and dysarthria. After undergoing a complete workup, the diagnosis of GBM was ultimately made. The patient underwent surgical resection followed by RT and chemotherapy. After developing new-onset neurological symptoms, she underwent repeat imaging and was subsequently diagnosed with disease recurrence. Given the tumor size, the patient was determined not to be a candidate for further surgical intervention. This case highlights the importance of a rapid and complete workup for patients who present with neurologic symptoms and further emphasizes the need for frequent surveillance and follow-up imaging to monitor for disease recurrence.
